# Comparative Genomics of 86 Whole-Genome Sequences in the Six Species of the *Elizabethkingia* Genus Reveals Intraspecific and Interspecific Divergence

**DOI:** 10.1038/s41598-019-55795-3

**Published:** 2019-12-16

**Authors:** Chih-Yu Liang, Chih-Hui Yang, Chung-Hsu Lai, Yi-Han Huang, Jiun-Nong Lin

**Affiliations:** 1 Department of Information Engineering, I-Shou University, Kaohsiung, Taiwan; 2 Department of Emergency Medicine, E-Da Cancer Hospital, I-Shou University, Kaohsiung, Taiwan; 3 Department of Medical Imaging and Radiological Science, College of Medicine, I-Shou University, Kaohsiung, 824 Taiwan; 4 Department of Biological Science and Technology, Meiho University, Pingtung, Taiwan; 5 School of Medicine, College of Medicine, I-Shou University, Kaohsiung, Taiwan; 6 Division of Infectious Diseases, Department of Internal Medicine, E-Da Hospital, I-Shou University, Kaohsiung, Taiwan; 7 Department of Critical Care Medicine, E-Da Hospital, I-Shou University, Kaohsiung, Taiwan

**Keywords:** Bacterial genomics, Bacterial infection

## Abstract

Bacteria of the genus *Elizabethkingia* are emerging infectious agents that can cause infection in humans. The number of published whole-genome sequences of *Elizabethkingia* is rapidly increasing. In this study, we used comparative genomics to investigate the genomes of the six species in the *Elizabethkingia* genus, namely *E. meningoseptica*, *E. anophelis*, *E. miricola*, *E. bruuniana*, *E. ursingii*, and *E. occulta*. *In silico* DNA–DNA hybridization, whole-genome sequence-based phylogeny, pan genome analysis, and Kyoto Encyclopedia of Genes and Genomes (KEGG) analyses were performed, and clusters of orthologous groups were evaluated. Of the 86 whole-genome sequences available in GenBank, 21 were complete genome sequences and 65 were shotgun sequences. *In silico* DNA–DNA hybridization clearly delineated the six *Elizabethkingia* species. Phylogenetic analysis confirmed that *E. bruuniana*, *E. ursingii*, and *E. occulta* were closer to *E. miricola* than to *E. meningoseptica* and *E. anophelis*. A total of 2,609 clusters of orthologous groups were identified among the six type strains of the *Elizabethkingia* genus. Metabolism-related clusters of orthologous groups accounted for the majority of gene families in KEGG analysis. New genes were identified that substantially increased the total repertoire of the pan genome after the addition of 86 *Elizabethkingia* genomes, which suggests that *Elizabethkingia* has shown adaptive evolution to environmental change. This study presents a comparative genomic analysis of *Elizabethkingia*, and the results of this study provide knowledge that facilitates a better understanding of this microorganism.

## Introduction

The species of the *Elizabethkingia* genus are usually found in natural environments, including water, soil, and plants, but they rarely cause diseases in humans^1–9^. The *Elizabethkingia* genus presently comprises six species. Among these, *E. meningoseptica*, initially named as *Flavobacterium meningosepticum*, was first identified by an American bacteriologist, Elizabeth O. King, in 1959^10^. This species was categorized into a new genus (i.e., the *Chryseobacterium* genus) and was renamed as *C. meningosepticum* in 1994^11^. With the application of 16 S ribosomal RNA (rRNA) in the taxonomy of microorganisms, *C. meningosepticum* and another novel species identified in 2003 (i.e., *C. miricola*) were assigned into a new genus (i.e., the *Elizabethkingia* genus) in 2005^12^. The third species, *E. anophelis*, was initially discovered from the midgut microbiota of the mosquito *Anopheles gambiae* in the Gambia, Africa, in 2011^13^. Lastly, three novel species, namely *E. bruuniana*, *E. ursingii*, and *E. occulta*, were proposed by Nicholson *et al*. in August 2017^14^.

Recently, *Elizabethkingia* infections in humans have been increasingly reported and emerged as a critical public health issue. Life-threatening infections caused by *E. anophelis* have been described in Africa, Singapore, Hong Kong, the USA, and Taiwan^2–9^. The most severe outbreak of *E. anophelis* reported on to date occurred in Wisconsin and Illinois between November 1, 2015 and April 12, 2017^5,7,9^.

With advances in molecular biology and biotechnology, whole-genome sequencing has become a popular technique in microbiology research. Whole-genome sequences of microbes can provide comprehensive information on virulence factors, pathogenesis, drug resistance, metabolism, host–pathogen interaction, host–environment reaction, and others. We previously published whole-genome sequences for two *Elizabethkingia* species: *E. anophelis* (strain EM361-97; GenBank accession number, LWDS00000000)^15^ and *E. miricola* (strain EM798-26; GenBank accession number, CP023746)^16^. *E. miricola* strain EM798-26 has been re-classified as *E. bruuniana* according to the whole-genome analysis^17,18^. However, few studies have investigated the comparative genomics of the six species in the *Elizabethkingia* genus. In this study, we used several methods to comprehensively analyze and compare the genomic features of all available whole-genome sequences of *Elizabethkingia* strains in GenBank.

## Materials and Methods

### Ethics and experimental biosafety statements

This study was approved by the Institutional Review Board of E-Da Hospital (EMRP-106-105). The need for patient’s informed consent was waived by the Institutional Review Board of E-Da Hospital as the retrospective analysis of clinical data posed no more than minimal risk of harm to subjects and involved no procedures for which written consent was normally required outside of the research context. The experiments in this study were approved by the Institutional Biosafety Committee of E-Da Hospital. All experiments were performed in accordance with relevant guidelines and regulations.

### Isolates for *g*enome *s*equencing

Two clinical strains, namely *E. anophelis* EM361-97 and *E. bruuniana* EM798-26, were isolated from the blood of two cancer patients in Taiwan. The detailed information has been described previously^15,16^. In brief, the genomic DNA of these two strains was sequenced using an Illumina HiSeq. 2000 sequencing platform (Illumina, San Diego, CA, USA), and the DNA short reads were assembled using SOAP^19^. The sequences of the *E. bruuniana* strain EM798-26 were further analyzed using PacBio (Pacific Biosciences of California, Menlo Park, CA, USA) and optical mapping (Bionano Genomics, San Diego, CA, USA). Finally, 26 scaffolds and 27 contigs were generated for the *E. anophelis* strain EM361-97^15^, and the complete whole-genome sequence of the *E. bruuniana* strain EM798-26 was constructed^16,17^. The gene annotations of these two strains were performed using the Prokaryotic Genome Annotation Pipeline of the National Center for Biotechnology Information (NCBI)^20^.

### Strains in this study

At the time of writing this paper, 86 whole-genome sequences of *Elizabethkingia* species, including 17 *E. meningoseptica* strains, 44 *E. anophelis* strains, 11 *E. miricola* strains, 8 *E. bruuniana* strains, 4 *E. ursingii* strains, and 2 *E. occulta* strains, were available in the NCBI genome sequence repository of GenBank (https://www.ncbi.nlm.nih.gov/genome/). Among these genomic sequences, 21 were complete genomes and 65 were shotgun sequences that presented as scaffolds or contigs. All these genome sequences were downloaded for comparison and analysis (Supplementary File 1).

### *In silico* DNA–DNA hybridization

For genome-based species delineation, *in silico* DNA–DNA hybridization (DDH) was performed using the Genome-to-Genome Distance Calculator (GGDC) (http://ggdc.dsmz.de/home.php)^21^. The results of formula 2 were adopted according to the suggestion of the authors in a previous study^21^. A cutoff value of 70% was used as the delimitation criteria of microorganism species^21^. The heat map was generated using CIMminer (https://discover.nci.nih.gov/cimminer/).

### Construction of whole-genome sequence-based phylogenetic tree

The whole-genome sequence-based phylogenetic tree was constructed using the Reference sequence Alignment based Phylogeny builder (REALPHY) (https://realphy.unibas.ch/fcgi/realphy)^22^. The whole-genome sequences of the 86 *Elizabethkingia* strains were submitted to the online pipeline of REALPHY in the FASTA format. The sequence alignments of phylogeny were performed using PhyML. The phylogenetic tree was edited using Dendroscope^23^.

### Analysis of orthologous genes

To evaluate the evolution of ancestor genes in the different species, the online program OrthoVenn (http://www.bioinfogenome.net/OrthoVenn/) was used to evaluate the clusters of orthologous groups (COGs) in the six type strains of the *Elizabethkingia* genus^24^. The whole-genome sequences of the six type strains, namely *E. meningoseptica* KC1913^T^ (=ATCC 13253^T^), *E. anophelis* R26^T^, *E. miricola* GTC 862^T^ (=KCTC 12492^T^ = W3-B1), *E. bruuniana* G0146^T^, *E. ursingii* G4122^T^, and *E. occulta* G4070^T^, were included for comparison of the COGs. The virulence factors were analysed using the Virulence Factor Database (VFDB)^25^. The antimicrobial resistance-associated genes and transposable element (transposon) genes were identified using the Rapid Annotations based on Subsystem Technology (RAST) Prokaryotic Genome Annotation Server (http://rast.nmpdr.org/)^26^.

### Pan genome analysis

To perform pan genome analysis of *Elizabethkingia* species, the core (conserved), accessory (dispensable), and unique (strain-specific) genes were analyzed using a software package, namely the Bacterial Pan Genome Analysis Tool (BPGA)^27^. The pan genome and core genome phylogenies were generated using BPGA with default settings as per the manufacturer’s instructions.

### Kyoto encyclopedia of genes and genomes (KEGG)

To understand the high-level functions of the *Elizabethkingia* strains, we used BPGA^27^ to access the KEGG database. The gene families of metabolism, genetic information processing, environmental information processing, cellular processes, organismal systems, human diseases, and drug development were analyzed.

## Results and Discussion

### Species delineation through *in silico* DDH

**Figure 1:**
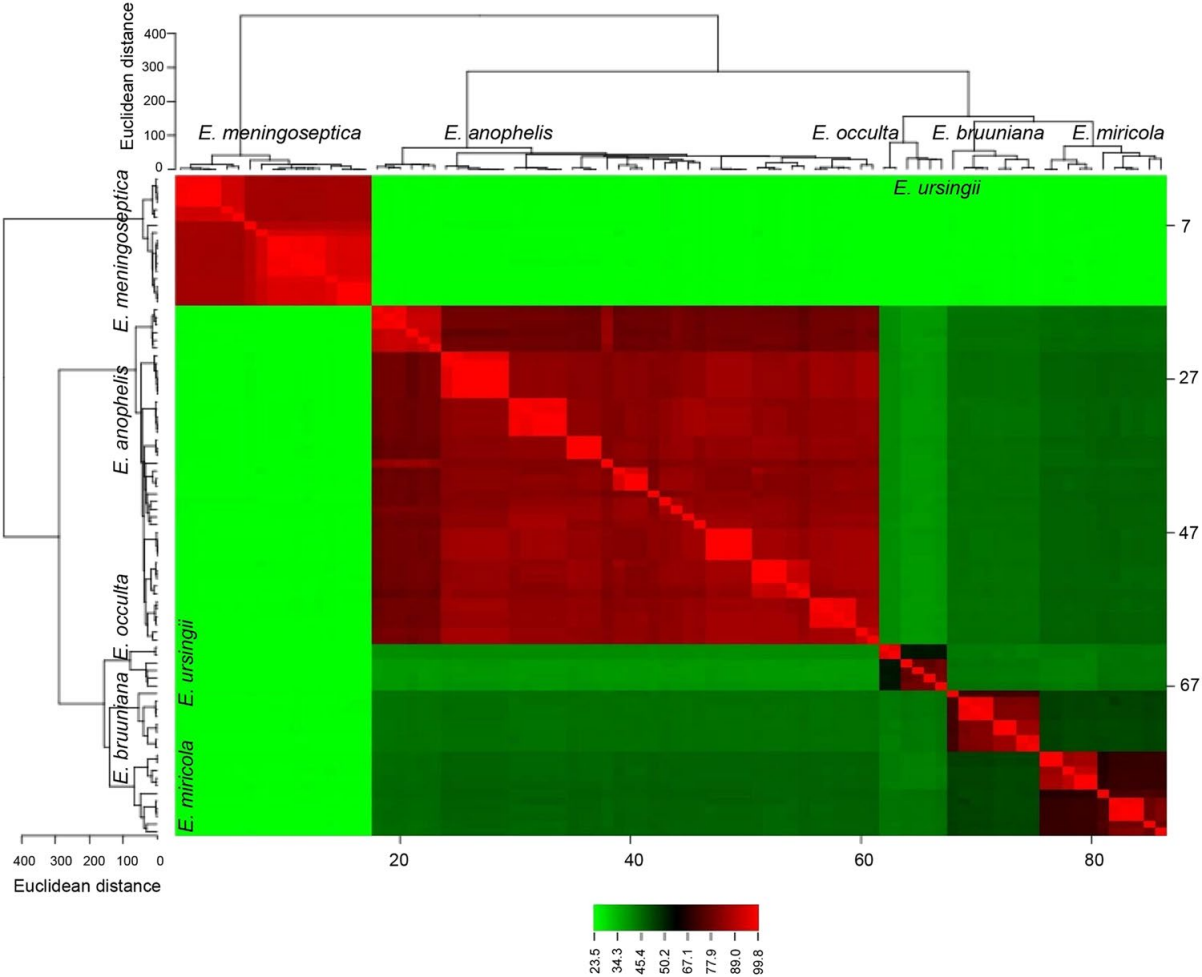
Dendrogram and heat map generated using *in silico* DNA–DNA hybridization (DDH) among 86 *Elizabethkingia* strains. The *in silico* DDH values between *E. meningoseptica* and the other five species were obviously lower than the values between the other species (light green). *E. bruuniana*, *E. ursingii*, and *E. occulta* were the most related to *E. miricola* in the dendrogram.

Figure 1 presents the results of *in silico* DDH for the 86 *Elizabethkingia* strains. The *in silico* DDH values between *E. meningoseptica* and the other five species were obviously lower than the values among the other species. The three novel species, namely *E. bruuniana*, *E. ursingii*, and *E. occulta*, were close to *E. miricola* in the dendrogram. The heat map built from the similarity matrix clearly displayed the delineation of the six species in the *Elizabethkingia* genus.

With the development of next-generation sequencing, numerous whole-genome sequences are available in the repositories of GenBank, and whole-genome sequencing can be used as a new method for species discrimination. For genome-based species delineation, *in silico* DDH is considered as an accurate substitution for traditional DDH^21,28–30^. Our study used GGDH, an *in silico* method for genome-to-genome comparison, to discriminate *Elizabethkingia* species. Similar to the results of the previous study performed by Nicholson *et al*.^14^, our study clearly supports that *in silico* DDH can noticeably distinguish *Elizabethkingia* species.

### Phylogenetic evolution based on whole genomes

**Figure 2:**
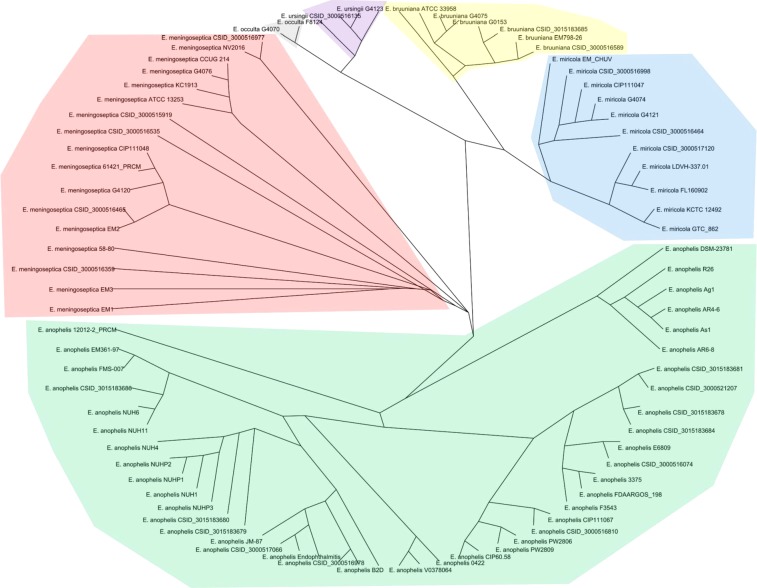
Phylogenetic evolution based on the whole-genome sequences of the 86 *Elizabethkingia* strains. The strains of the six *Elizabethkingia* species were separately clustered.

Figure 2 illustrates the phylogenetic tree based on the whole-genome sequences of the 86 *Elizabethkingia* strains, which was constructed using REALPHY. The six species of the *Elizabethkingia* genus were evidently separate from each other. Similar to the dendrogram generated based on *in silico* DDH, *E. bruuniana*, *E. ursingii*, and *E. occulta* were located close to *E. miricola* and were away from *E. anophelis* and *E. meningoseptica*. *E. anophelis* were divided into several sublineages.

Phylogeny constructed using whole-genome sequences is known to be more congruent than that built using a single gene or a small fraction of the genome, such as 16S rRNA or *rpoB* genes^31^. Through traditional DDH, *Elizabethkingia* species were previously classified as genomospecies. Based on the phylogenetic study of whole-genome sequences, *E. miricola* was found to be the most similar to genomospecies 2, *E. anophelis* was the closest to genomospecies 1, genomospecies 3 was near *E. bruuniana*, and genomospecies 4 was proposed as *E. ursingii*^14^. Moreover, the phylogenetic tree of *E. anophelis* strains showed several clearly demarcated sublineages in our study. The analysis of genomic features in the Wisconsin outbreak of *E. anophelis* also revealed different phylogenetic subclusters with distinctive temporal and geographic dynamics^5^. In the present study, we constructed the phylogenetic tree of the 86 *Elizabethkingia* strains using whole-genome sequences; the phylogenetic tree clearly demonstrated the phylogenetic relationship among these strains. The phylogeny generated using whole genomes provides not only species delineation but also comprehensive insights into comparative analyses of phylogenetic evolution across different species.

### Functional COGs

COGs, also known as orthologs, are a group of genes in different species that have descended from a common gene in the same ancestor^32^. These genes usually retain the original function during the evolution of microorganisms, and they determine the relationships between the genome structure, gene function, and taxonomic classification^32^. Subsequently, it is crucial to recognize COGs and predict their functions, particularly in emerging pathogens with newly sequenced genomes^32^.

**Figure 3:**
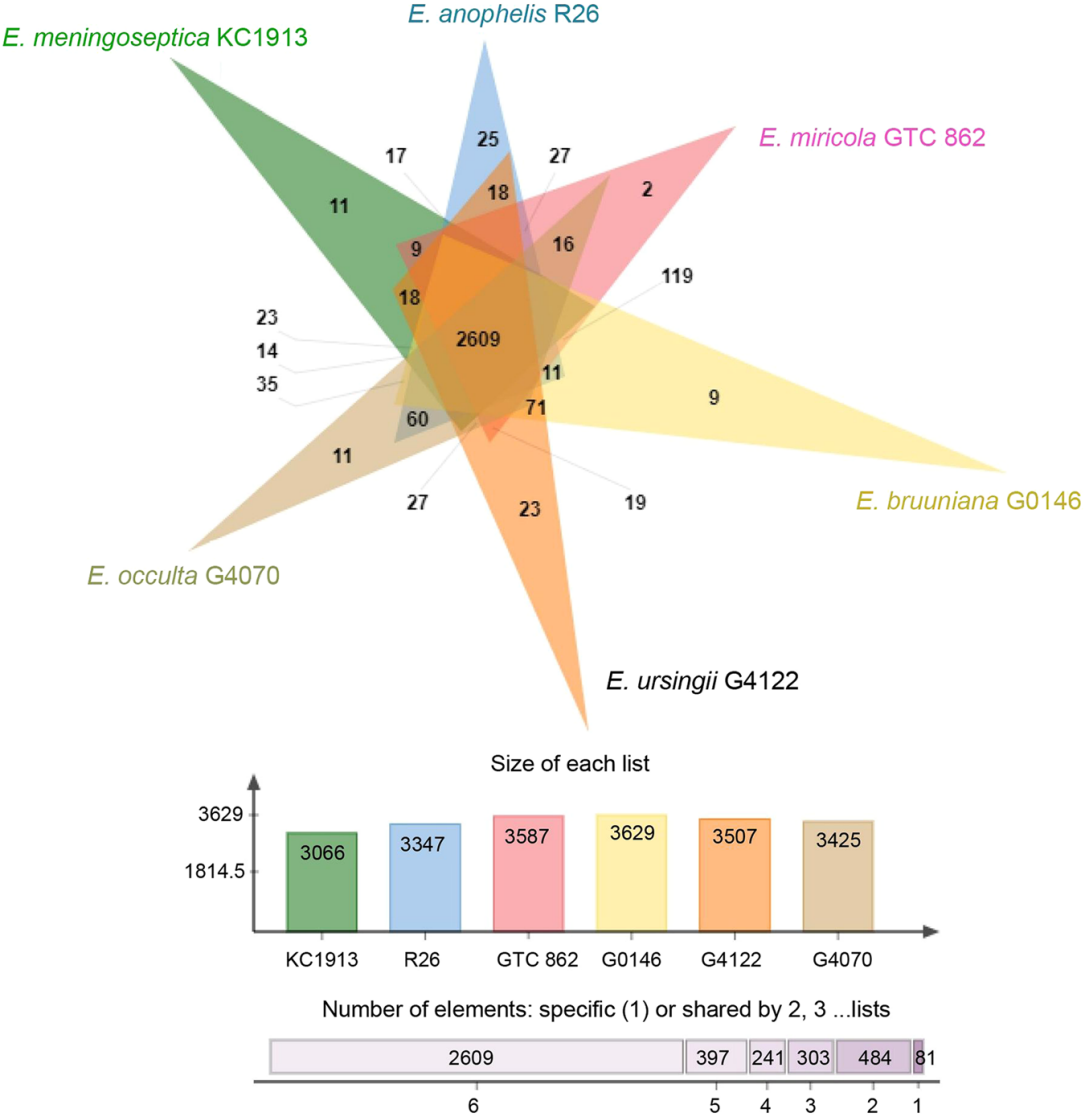
Venn diagram illustrating the distribution of shared and specific clusters of orthologous groups in the six type strains of the *Elizabethkingia* genus. A total of 2,609 shared clusters of orthologous groups were identified in these six type strains.

Figure 3 presents the COGs of the six *Elizabethkingia* type species. The total number of genes in the six species ranged from 3,066 to 3,629. The *E. bruuniana* strain G0146 possessed the highest number of genes, and the *E. meningoseptica* strain KC1913 had the lowest number of genes. A total of 2,609 shared COGs were identified among the six *Elizabethkingia* type species (Fig. 3; Supplementary File 2). The *E. miricola* strain GTC 862 had the lowest number of unique gene families (n = 2), and the *E. anophelis* strain R26 had the highest number of unique gene families (n = 25). The difference in the number of unique gene families may reflect the phenotypical traits that are specific to the group of bacteria^33^.

In the present study, the genome annotation of the six *Elizabethkingia* type species using the RAST Server revealed abundant putative genes associated with transposable elements. These transposons included hypothetical conjugative transposons BF0131, putative conjugative transposon mobilization protein BF0132, putative mobilization protein BF0133, and conjugative transposon protein TraA, TraB, TraE, TraF, TraG, TraJ, TraK, TraM, TraN, TraO, and TraQ. A recent study investigated putative integrative and conjugative elements (ICEs) in 13 complete genomes and 23 draft genomes of *E. anophelis* strains^34^. Among the 36 *E. anophelis* strains, ICEs were identified in 31 strains. ICEs are an important group of mobile genetic elements, which could transfer between bacteria horizontally via conjugation^34^. However, further studies are necessary to investigate if horizontal gene transfer occurs between different strains of *Elizabethkingia*.

Patients infected with *Elizabethkingia* have shown a mortality rate of 34% to 60%. The immune status of patients and virulence factors of microorganisms may be associated with the high case-fatality rate of *Elizabethkingia* infections. Previous studies have shown that patients with *Elizabethkingia* infections frequently had chronic illnesses. For example, 85% of patients with *E. anophelis* infections had comorbidities, such as malignancy (45%), cardiovascular diseases (37%), and diabetes mellitus (25%)^6^. In the present study, many homologs of offensive, defensive, nonspecific, and virulence-associated regulatory factors were identified in the COGs of *Elizabethkingia* isolates (Supplementary File 2). The potential virulence factor homologs and their associated genes of the six *Elizabethkingia* species predicted using the VFDB are shown in Table 1. These virulence factors included heat shock protein, phospholipase, capsular polysaccharide, catalase, peroxidase, and others. The distribution of virulence factors was similar among *E. meningoseptica* KC1913^T^, *E. anophelis* R26^T^, and *E. bruuniana* G0146^T^. Some virulence factors, such as polar flagella (*flmH*), exopolysaccharide (*galE*, *pgi*, *acpXL*, *hemL*, *ureB*, *ureG*), Mg^2+^ transport (*mgtB*), and type III secretion system effectors (*hopJ1*) were identified only in *E. miricola* GTC 862^T^, *E. ursingii* G4122^T^, and *E. occulta* G4070^T^. It is interesting that there are no flagella in *Elizabethkingia* species. However, the putative *flmH* was identified in *E. miricola*, *E. ursingii*, and *E. occulta*

**Table 1:** The potential virulence factor homologs and their associated genes of the six *Elizabethkingia* species predicted using the Virulence Factor Database (VFDB).

Classification of Virulence factors	Virulence Factors	*E. meningoseptica* KC1913	*E. miricola* GTC 862	*E. anophelis* R26	*E. bruuniana* G0146	*E. ursingii* G4122	*E. occulta* G4070
Adherence	Hsp60	*htpB*	*htpB*	*htpB*	*htpB*	*htpB*	*htpB*
	Polar flagella	—	*flmH*	—	—	*flmH*	*flmH*
Biofilm formation	AdeFGH efflux pump/transport autoinducer	*htpB*	—	—	—	—	—
		—	*adeG*	*adeG*	*adeG*	*adeG*	*adeG*
Enzyme	Phospholipase C	*plc*	*plc*	*plc*	*plc*	*plc*	*plc*
	Phospholipase D	—	—	—	—	—	—
	Streptococcal enolase	*eno*	*eno*	*eno*	*eno*	*eno*	*eno*
Immune evasion	Exopolysaccharide	—	*galE*	—	—	*galE*	*galE*
		—	*pgi*	—	—	*pgi*	*pgi*
	LPS	—	*acpXL*	—	—	*acpXL*	*acpXL*
Iron uptake	Heme biosynthesis	—	*hemL*	—	—	*hemL*	*hemL*
Acid resistance	Urease	—	*ureB*	—	—	*ureB*	*ureB*
		—	*ureG*	—	—	*ureG*	*ureG*
Lipid and fatty acid metabolism	Isocitrate lyase	*icl*	*icl*	*icl*	*icl*	*icl*	*icl*
	Pantothenate synthesis	*panD*	*panD*	*panD*	*panD*	*panD*	*panD*
Magnesium uptake	Mg2 + transport	—	*mgtB*	—	—	*mgtB*	*mgtB*
Other adhesion-related proteins	EF-Tu	*tuf*	*tuf*	*tuf*	*tuf*	*tuf*	*tuf*
Secretion system	Type III secretion system effectors	—	*hopJ1*	—	—	*hopJ1*	*hopJ1*
Stress adaptation	Catalase-peroxidase	*katG*	*katG*	*katG*	*katG*	*katG*	*katG*
	Catalase	*katA*	*katA*	*katA*	*katA*	*katA*	*katA*
Macrophage inducible genes	Mig-5	*mig*-5	—	—	—	—	—
Serum resistance and immune evasion	Lipopolysaccharide	—	—	—	—	—	*wbt*I
	O-antigen	—	—	—	—	*fcl*	*fcl*

. Further studies are necessary to investigate the function and source of this putative gene.

**Figure 4:**
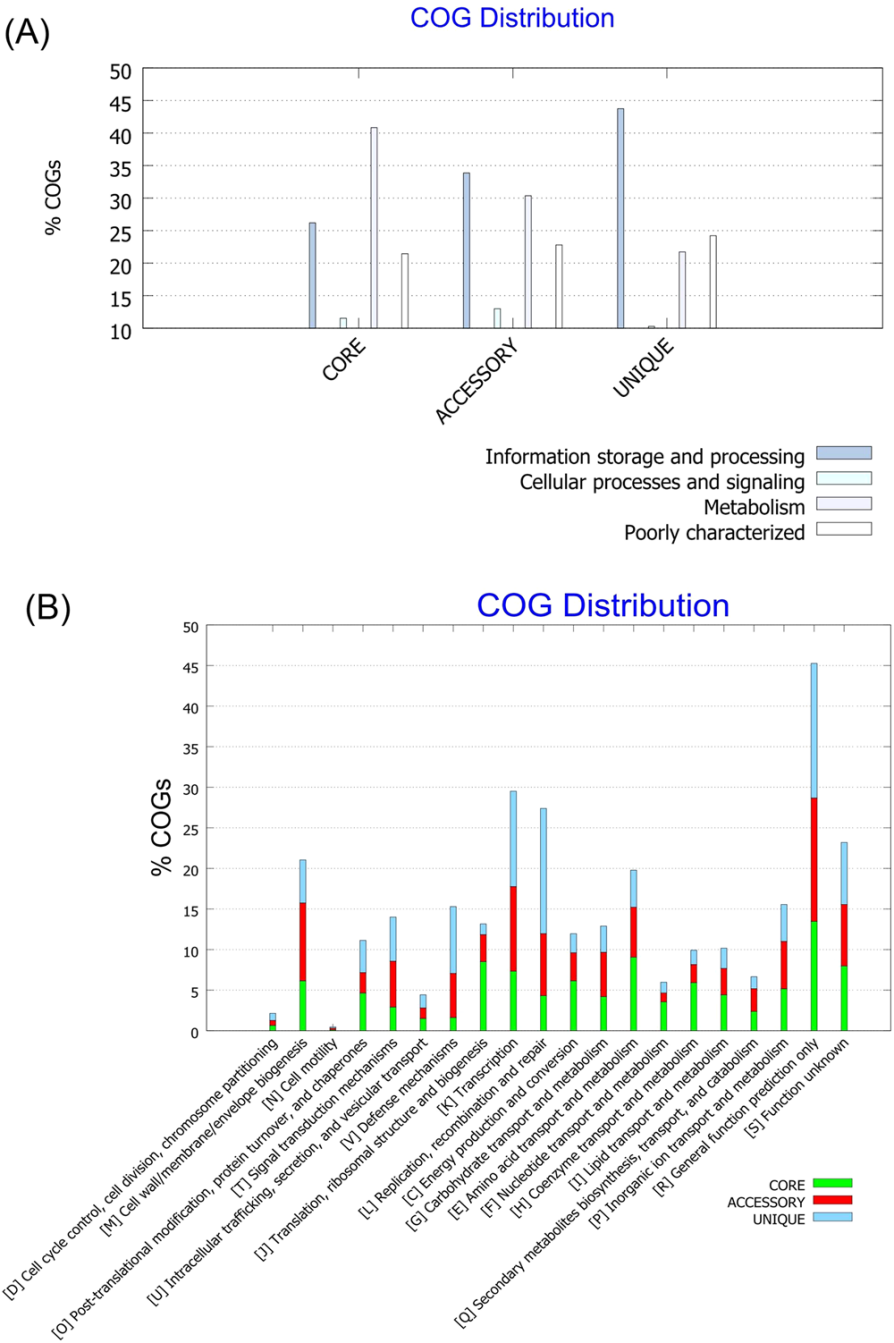
Clusters of orthologous groups (COGs) in core, accessory, and unique genomes and their associated functions. (**A**) Distribution of functional COGs in each core, accessory, and unique genome. (**B**) Detailed distribution of COGs with their functions.

Functional analysis of the COGs in the 86 *Elizabethkingia* genomes revealed that the majority of core genomes were associated with metabolism, and the unique gene families were mostly related to “information storage and processing” (Fig. 4A). In the COG analysis, “information storage and processing” includes RNA processing and modification, chromosome dynamics, translation, transcription, replication, recombination, and repair^35^. The function of COGs with “information storage and processing” might be associated with intracellular survival^36^. However, the exact reason for the large presence of genes with function of “information storage and processing” in unique gene families is not clear.

According to the functional prediction of genomes, R (general function prediction only) accounted for the largest part of COGs, followed by K (transcription) and L (replication, recombination, and repair). Gene families associated with D (cell cycle control, cell division, and chromosome partitioning) occupied the least part (Fig. 4B). Regarding the constituents of each functional gene family, core genomes consisted of 64.7% of J (translation, ribosomal structure, and biogenesis), and accessory genomes accounted for the largest part of M (cell wall, membrane, envelope, and biogenesis) (45.7%). The unique genes accounted for 56.3% and 53.7% of L (replication, recombination, and repair) and V (defense mechanisms), respectively.

### KEGG

**Figure 5:**
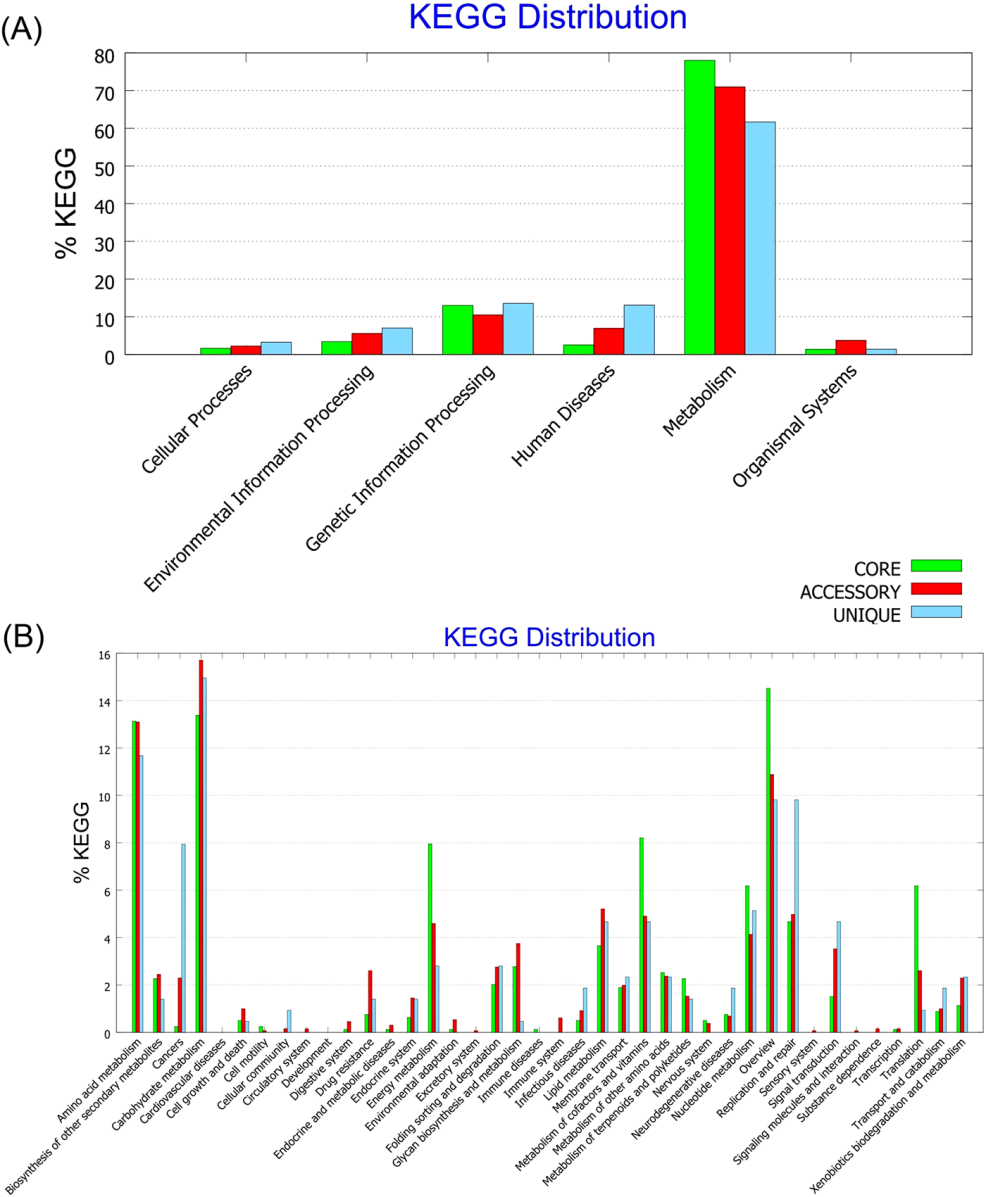
Kyoto Encyclopedia of Genes and Genomes (KEGG) analysis of the 86 *Elizabethkingia* strains. (**A**) The majority of core, accessory, and unique genes were associated with metabolism. (**B**) Functional annotations showed that gene families associated with carbohydrate metabolism, amino acid metabolism, cofactor and vitamin metabolism, and energy metabolism accounted for the largest part in these 86 *Elizabethkingia* strains.

In the KEGG analysis, genes associated with metabolism accounted for the largest part (Fig. 5A). Of these genes, most were associated with carbohydrate metabolism, followed by amino acid metabolism, cofactor and vitamin metabolism, and energy metabolism (Fig. 5B). For genes associated with carbohydrate metabolism, core genes accounted for 30.4%, accessory genes for 35.7%, and unique genes for 33.9%.

The antimicrobial resistance-associated proteins in the six *Elizabethkingia* type species are shown in Table 2. Multidrug resistance efflux pumps, β-lactamases, proteins associated with resistance to vancomycin, and quinolone-resistance determining regions (DNA gyrase and topoisomerase IV) were extensively identified in these species. Several antimicrobial resistance-associated proteins, such as multidrug resistance efflux pumps, multiple antibiotic resistance MAR locus, and multidrug resistance, tripartite systems, were identified in some species. No antimicrobial resistance-associated genes were identified on ICEs in the genome annotation using the RAST Server.

**Table 2:** The antimicrobial resistance-associated proteins of the six *Elizabethkingia* type species identified using the Rapid Annotations based on Subsystem Technology (RAST) Prokaryotic Genome Annotation Server.

Class of Antimicrobial Resistance	E. meningoseptica KC1913	E. miricola GTC 862	E. anophelis R26	E. bruuniana G0146	E. ursingii G4122	E. occulta G4070
β-lactamase	β-lactamase	β-lactamase	β-lactamase	β-lactamase	β-lactamase	β-lactamase
	BLI*	BLI	BLI	BLI	BLI	BLI
	—	—	—	β-lactamase Class C	—	—
Multidrug resistance efflux pumps	CmeB	CmeB	CmeB	CmeB	—	—
	—	TolC	—	TolC	TolC	—
	MATE family MDR Pump	MATE family MDR Pump	MATE family MDR Pump	MATE family MDR Pump	MATE family MDR Pump	MATE family MDR Pump
	OML	OML	OML	OML	OML	OML
	AcrB	AcrB	AcrB	AcrB	AcrB	AcrB
Multiple antibiotic resistance MAR locus	—	MarA, MarB	—	MarA, MarB	MarC	MarC
Multidrug resistance, tripartite systems	MFP	MFP	MFP	MFP	—	—
	IM	IM	IM	IM	—	—
	OM	OM	OM	OM	—	—
Resistance to vancomycin	VanW	VanW	VanW	VanW	VanW	VanW
Resistance to fluoroquinolones–DNA gyrase	GyrA, GyrB	GyrA, GyrB	GyrA, GyrB	GyrA, GyrB	GyrA, GyrB	GyrA, GyrB
Topoisomerase IV	ParC, ParE	ParC, ParE	ParC, ParE	ParC, ParE	ParC, ParE	ParC, ParE

*BLI: metal-dependent hydrolases of the beta-lactamase superfamily I.

Previous studies have shown that *Elizabethkingia* isolates were frequently resistant to many antimicrobial agents, including most β-lactams, β-lactams/β-lactamase inhibitors, aminoglycosides, macrolides, tetracycline, vancomycin, and carbapenems. But they showed variable susceptibility to piperacillin, piperacillin-tazobactam, fluoroquinolones, minocycline, tigecycline, and trimethoprim-sulfamethoxazole^2–6,37^. Genes associated with drug resistance in the *Elizabethkingia* genus have been reported. For example, Opota *et al*. reported that *bla*_*GOB-13*_ and *bla*_*B-9*_ carbapenemase-encoding genes were identified in a carbapenemase-producing clinical isolate, *Elizabethkingia miricola* EM_CHUV^38^. Previous reports indicated that *E. meningoseptica* and *E. anophelis* were resistant to several classes of antimicrobials^2–6^. A recent study revealed that *E. bruuniana* has a similar antimicrobial susceptibility pattern to other *Elizabethkingia* species^18^. The present study also showed that the antimicrobial resistance-associated genes of the three new *Elizabethkingia* species are similar to those of the other three species.

It is noteworthy that vancomycin resistance gene (*vanW*) was found in these six *Elizabethkingia* type species. *vanW* is included in the *vanB* gene cluster. The exact function of *vanW* is still unknown. However, mutations in *vanW* has been identified in microorganisms with VanB-type glycopeptide resistance^39^. Vancomycin has been anecdotally reported to successfully treat patients with *E. meningoseptica* meningitis^40^. However, several recent studies revealed that most *Elizabethkingia* species exhibited a high minimum inhibitory concentration of vancomycin^41,42^. Therefore, the use of vancomycin is not suggested for patients with *Elizabethkingia* infections^41,42^.

### Core and pan genome analysis

**Figure 6:**
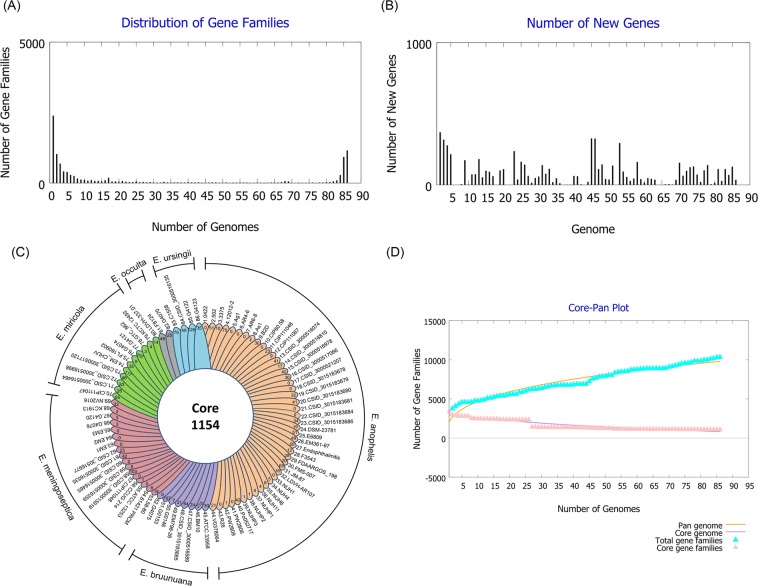
Pan genome analysis of 86 *Elizabethkingia* strains. (**A**) Distribution of gene families among the 86 *Elizabethkingia* strains. (**B**) Distribution of new genes among the 86 *Elizabethkingia* strains. (**C**) Flower plot showing the numbers of core orthologous genes (in the center) and strain-specific orthologous genes (in the petals) in each *Elizabethkingia* strain. (**D**) Core-pan plot of the 86 *Elizabethkingia* strains. The orange and purple lines indicate the numbers of pan genomes and core genomes, respectively. The cyan lines represent the change in gene families in pan genomes when genomes were added sequentially. This curve of pan genome denotes that the pan genome is still open after adding 86 genomes. The pink lines indicate the change in gene families in core genomes when genomes were added sequentially.

In 2005, Tettelin *et al*. proposed pan genome as the whole-genomic repertoire of a microorganism^43^. Pan genome analysis can be used to discriminate the diversity of genomes and explore the core, accessory, and unique genes^44^. To understand the pan genome of *Elizabethkingia*, the whole-genome sequences of the 86 strains were examined. The distribution of gene families and the number of new genes are shown in Fig. 6A,B, respectively. The core, accessory, and unique genes are presented as a flower plot in Fig. 6C. In the 86 *Elizabethkingia* strains, 1,154 core (conserved) genes were recognized. In each strain, the number of accessory genes ranged from 996 to 2,738, and the number of unique genes ranged from 0 to 215. With the addition of new genome sequences, the genes of the pan genome increased from 2,110 to 9,794, and core genes decreased from 3,002 to 824 (Fig. 6D).

## Conclusions

This study presents a comparative genomic analysis of 86 *Elizabethkingia* strains with whole-genome sequences available in GenBank. Because *Elizabethkingia* infections have emerged as a critical public health issue worldwide, knowledge on the clinical, molecular, and genetic characteristics is of paramount importance. The results of this study provide information to understand the population genomics, phylogenetic distinctness, evolutionary features, and genetic functions of this emerging and life-threatening pathogen.

## Supplementary information

Supplementary File 1

Supplementary File 2

## Data Availability

The names of organisms, strains, biosample numbers, bioproject numbers, assembly numbers, isolated origins, and release dates of bacteria used in this study are shown in Supplementary File 1. All data are available in the NCBI genome sequence repository.
